# Stereo Event-Based Visual–Inertial Odometry

**DOI:** 10.3390/s25030887

**Published:** 2025-01-31

**Authors:** Kunfeng Wang, Kaichun Zhao, Wenshuai Lu, Zheng You

**Affiliations:** Department of Precision Instrument, Tsinghua University, Beijing 100080, China; wkf18@mails.tsing-hua.edu.cn (K.W.); kaichunz@mail.tsinghua.edu.cn (K.Z.); luwenshuai_dky@163.com (W.L.)

**Keywords:** event-based camera, visual–inertial odometry, ESKF

## Abstract

Event-based cameras are a new type of vision sensor in which pixels operate independently and respond asynchronously to changes in brightness with microsecond resolution, instead of providing standard intensity frames. Compared with traditional cameras, event-based cameras have low latency, no motion blur, and high dynamic range (HDR), which provide possibilities for robots to deal with some challenging scenes. We propose a visual–inertial odometry for stereo event-based cameras based on Error-State Kalman Filter (ESKF). The vision module updates the pose by relying on the edge alignment of a semi-dense 3D map to a 2D image, while the IMU module updates the pose using median integration. We evaluate our method on public datasets with general 6-DoF motion (three-axis translation and three-axis rotation) and compare the results against the ground truth. We compared our results with those from other methods, demonstrating the effectiveness of our approach.

## 1. Introduction

Simultaneous localization and mapping (SLAM) has important applications in many emerging technologies such as robotics, intelligent transportation, and augmented/virtual reality (AR/VR). There are already many works on SLAM based on traditional cameras [1,2]. However, traditional cameras can struggle in challenging situations, such as capturing high-speed motions or scenes with a high dynamic range.

Event-based cameras (or event cameras) are bio-inspired vision sensors that work very different from traditional cameras which report the pixel-wise intensity changes asynchronously at the time they occur, called “events” [3,4], where each event consists of its spatio-temporal coordinate and the sign of the brightness change (e.g., 0 or 1). Event cameras have different types of sensors, such as Dynamic Vision Sensor (DVS) [3], DAVIS or ATIS. They do not output an intensity image at a fixed rate but a stream of asynchronous events at microsecond resolution. Event cameras offer numerous advantages over traditional cameras, including microsecond latency, low power consumption, and high dynamic range (e.g., 140 dB). With microsecond resolution, event cameras can work at high-speed motions, which can cause blurry images from traditional cameras.

The main challenge is how to make event cameras applicable to SLAM and other vision tasks [4]. Because the output of event cameras is different from traditional cameras, vision algorithms designed for traditional cameras cannot be directly applied to event cameras. Thus, we need new methods to process the data from these novel cameras. There are already some works for event cameras, such as feature tracking [5,6], 3D orientation estimation [7,8,9,10], and simultaneous localization and mapping (SLAM) [11,12,13,14,15,16].

**Figure 1:**
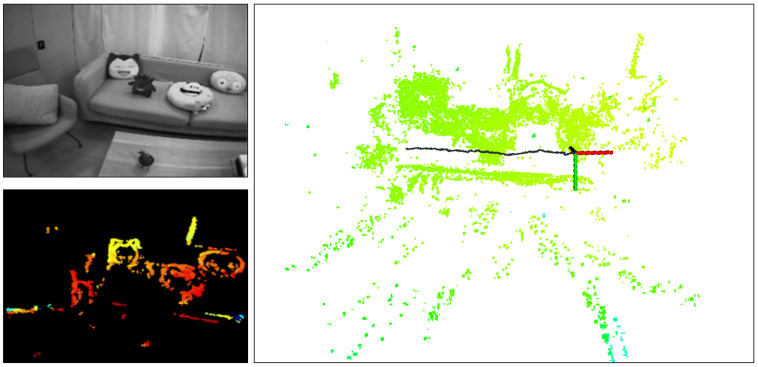
(**Top left**): scene. (**Bottom left**): inverse depth map at time *t*, and different colors represent different depths. (**Right**): global map and pose estimation.

Due to the limitations in accuracy and robustness when using a single sensor for localization, an IMU is typically used as a complementary sensor when employing cameras for positioning. Current sensor fusion methods can be broadly classified into two types: optimization-based and filtering-based. Generally, optimization-based methods require higher computational power, while filtering-based methods demand less computational effort but may offer slightly lower accuracy. Given that several optimization-based fusion approaches have already been proposed, we introduce a filtering-based fusion scheme, aiming to provide a new solution for stereo event camera–IMU fusion. In this paper, we propose a stereo visual–inertial odometry (VIO) for event cameras with arbitrary 6-DoF motion. As shown in Figure 1, the upper left corner displays an image captured by a traditional camera, which does not contribute to our algorithm. The image on the right illustrates the running performance of our algorithm, including local point cloud and trajectory (shown as black line). Our pipeline has three parts: vision module, IMU integral, and ESKF. For the vision module, we reference the strategy of ESVO [16]. IMU data use the median integral, and the fusion strategy is ESKF. Our work can be summarized as follows:

- A novel visual–inertial odometry is presented for stereo event cameras based on ESKF.
- Our method relies solely on visual information from event cameras and does not incorporate traditional cameras. Some approaches use images from traditional cameras, and in certain challenging scenarios, the failure of the traditional camera can lead to system failure, negatively impacting the performance of the event camera.
- A quantitative evaluation of our pipeline is compared with other methods on the public event camera datasets, demonstrating the effectiveness of our system.

## 2. Related Work

In the past few years, many works have been proposed to use event cameras for ego-motion estimation. Here, we review some of the literature [4].

### 2.1. Event-Based Depth Estimation

(a) Monocular: Depth estimation using a single event camera has been explored in studies such as [11,17,18]. This problem differs significantly from previous approaches due to the challenge of matching events over time. These methods produce a semi-dense 3D point cloud (referred to as a 3D edge map) of the scene, leveraging the camera’s motion information over time. Thus, they are not designed for instantaneous depth estimation but rather concentrate on depth estimation for SLAM/VO applications.

(b) Stereo: Lots of studies on depth estimation by event cameras utilize events over very short time periods, from two or more synchronized event cameras which are rigidly attached. Events from different event camera imaging planes use a common clock. According to the classic depth estimation method, the process first involves matching events across image planes and then triangulating the location of the 3D points [19]. The challenge is to find correlations between events at different moments. Events are matched either (i) using traditional stereo vision matching metrics (e.g., normalized cross-correlation) on event frames [20,21] or time-surfaces [22,23], or (ii) by exploiting the temporal correlations of events between different sensors [22,24,25]. Most of these methods are suitable for simple scenes and few objects, which facilitates finding correspondences.

### 2.2. Event-Based 3-DOF Estimation

The 3-DOF estimation includes rotation [7,8,9,10] and planar motion [26].

Cook et al. [7] proposed an algorithm that relies on an event camera to estimate ego motion, image intensity, and optical flow with an interacting network. However, it was only applied to pure rotational motion. The filter-based approach in [8] used probabilistic filters in parallel to track event camera’s three-degree-of-freedom rotation, creating images of natural scenes. Rotational motion estimation was also implemented in [9], where camera pose estimation was achieved by minimizing the photometric error at the event locations with a probabilistic edge map. A motion compensation optimization framework was introduced in [10] to estimate the angular velocity of the camera rather than its absolute rotation. All of the above works are limited to rotation estimation, not translation.

Weikersdorfer et al. [26] proposed a 2D SLAM system based on an event camera, which was limited to planar motion and scenes with rich textures. This method employed a particle filter for pose estimation, with the reprojection error of the event relative to the local map.

### 2.3. Event-Based VO

Solutions for 3D SLAM in any 6-DOF motion and scenes, with pure event cameras, have been proposed [11,12,13,14,15,16].

(a) Monocular: The approach in [11] extended [8] by using three probabilistic filters to estimate pose, depths of events and intensity images. However, it consumed a lot of computing power and required a GPU to run in real-time. In contrast, the semi-dense approach in [12] demonstrated that intensity image reconstruction was unnecessary for depth estimation and pose estimation. This method performed 3D reconstruction by space sweeping [17] and edge-map alignment for pose estimation. The final SLAM system could run in real-time on a CPU. The formulation in [13] was underpinned by a principled joint optimization problem involving non-parametric Gaussian Process motion modeling and incremental maximum a posteriori inference. However, its computational power consumption was also high, making it difficult to achieve real-time performance. Chamorro et al. [14] explored a new event-based line-SLAM approach following a parallel tracking and mapping design. Wang et al. [15] proposed a solution to this problem by performing contrast maximization in 3D. The 3D locations of the rays cast for each event were smoothly varied based on a continuous-time motion parametrization, and the optimal results were determined by maximizing the contrast in a volumetric ray density field.

(b) Stereo: The approach proposed in [16] addressed the solution for stereo visual odometry based on event cameras, achieving real-time performance with a standard CPU. It reconstructed a semi-dense 3D map following two steps: (i) computing depth estimates of events and (ii) fusing these depth estimates to obtain a more accurate semi-dense depth map [23].

### 2.4. Event-Based VIO

To improve the accuracy and robustness of visual odometry, combining with other sensors is a common option, such as IMU.

Zhu et al. [27] tracked features using [5], and combined them with IMU data by way of a Kalman filter. Rebecq et al. [28] proposed to synthesize motion compensated event images from the spatio-temporal windows of events and then detect-and-track features. Feature tracking was fused with inertial data by means of keyframe-based nonlinear optimization to recover the camera trajectory and a sparse map of 3D landmarks. Based on this work, the laboratory subsequently expanded to integrate event camera with traditional camera and IMU [29]. As opposed to the above-mentioned feature-based methods, the work in [30] optimized a combined objective with inertial and event-reprojection error terms over a segment of the camera trajectory, in the style of visual–inertial bundle adjustment. Gentil et al. [31] introduced an optimization-based framework using Lines. Liu et al. [32] fused the events’ depth, time-surface images, and pre-integrated data to estimate the camera motion within a sliding window nonlinear optimization framework. There are also some works that combine images, events, and IMU data, such as [29,33]. However, we think that the advantage of event cameras lies in their ability to handle high-speed motion or high dynamic range scenes. While integrating event cameras with traditional cameras may offer benefits, in certain challenging scenarios, the failure of the traditional camera can cause the entire system to fail, which can ultimately limit the performance of the event camera.

## 3. Visual–Inertial Pipeline

This section presents the details of stereo event-based visual–inertial odometry. We start with an overview of the entire pipeline. Then, we introduce how to process event data so that we can use their information efficiently. After that, we give the details of the vision module and IMU integral module. At least, we present how to fuse information from different sensors with ESKF.

### 3.1. Framework Overview

**Figure 2:**
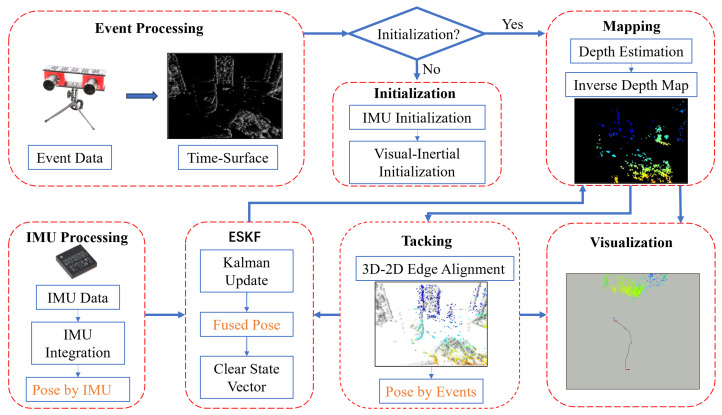
Overview of our proposed stereo event-based visual–inertial odometry.

A flowchart of our proposed pipeline detailing all steps is illustrated in Figure 2. We propose a novel method for fusing event camera data with IMU data to achieve localization based on ESKF. Our algorithm utilizes three parallel threads: the IMU integration thread, the mapping thread, and the tracking thread. We can obtain the current pose from both the IMU and the stereo event cameras, and then fuse these two poses using ESKF to obtain the final fused pose. In the ESKF framework, state variables require both predictive and observational information. The IMU provides the predictive information, while the visual module provides the observational information. Through the IMU integration thread, we obtain the current pose (pose by IMU), which serves as the predictive or prior information. The tracking thread in the visual module also provides the current pose (pose by events), which we use as the observation. When constructing the local point cloud map, we fuse the pose obtained from the IMU with the pose from the tracking thread using ESKF, then we obtain the current fused pose and the final pose output of the system.

Next, we provide a brief overview of the content covered in each section of this section. First, the event preprocessing module converts event streams into time-surface images, which are used by the vision module (Section 3.2). Secondly, we explain how the visual module obtains poses using data from the stereo event cameras. After initialization is completed, the mapping thread of the vision module uses the time-surfaces, events and the current pose (fused pose) at this moment to update a local map (semi-dense point cloud). The tracking thread of the vision module estimates the pose of the left event camera by aligning the local point cloud with the time-surface image (Section 3.3). Finally, we introduce the IMU integration process and the implementation of ESKF (Section 3.4).

Initialization: The vision module provides a coarse initial map by a stereo method (a modified SGM method [34]). The IMU module assumes that the initial state of the system is static [35,36], using the first 1–2 s (depending on dataset) of the IMU data to estimate the gravity direction and the biases of the gyroscope and accelerometer.

### 3.2. Event Representation (Time-Surface)

The output of the event camera is a number of independent events. Each event can be described as $e_{k}=\left(u_{k},v_{k},t_{k},p_{k}\right)$, includes pixel coordinate ${\left(u_{k},v_{k}\right)}^{T}$, where intensity is changed beyond the preset value, timestamp $t_{k}$, and polarity $p_{k}\left(\left\{-1,1\right\}\;or\;\left\{0,1\right\}\right)$.

**Figure 3:**
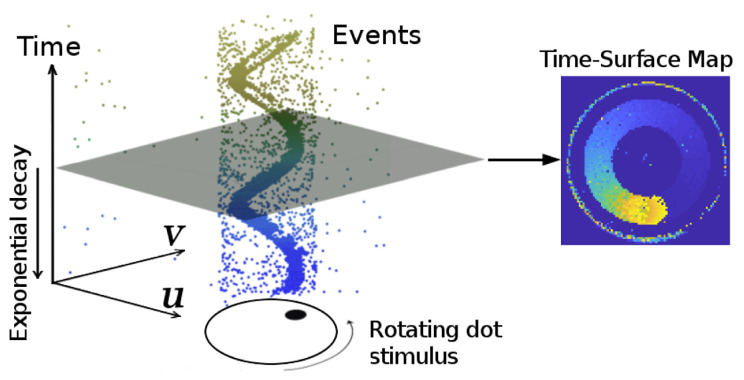
Time-surface. (**Left**): output of an event camera, and different colors represent different times. (**Right**): time-surface map. Figure adapted from [16].

Generally, we do not process events asynchronously at the very high rate they occur, but use an alternative representation called the time-surface (shown in Figure 3). It is a 2D image, and each pixel stores a value of time, which represents the timestamp of the last event at this pixel [37,38]. This converts events into an image whose ‘intensity’ represents the motion history at that coordinate, with larger values indicating more recent motion history. The value at each pixel **x** = ${\left(u,v\right)}^{T}$ is defined by

(1) $$\Gamma ≐exp(-\frac{t-t_{last}\left(x\right)}{\eta })$$

where *t* is an arbitrary time $\left(t>t_{last}\left(x\right)\right)$, and $t_{last}\left(x\right)$ is the timestamp of the last event occurring at x. η denotes the constant decay rate parameter, which usually is small. The time-surface visualizes the history of moving brightness change at each pixel location, which usually presents the edges of the scenes. The pixel values in a time-surface are rescaled from [0, 1] to the range [0, 255] for convenient visualization and processing.

### 3.3. Vision Module

This module is mainly divided into two parts: mapping (Section 3.3.1) and tracking (Section 3.3.2). The mapping and tracking threads operate simultaneously and are interdependent. The tracking thread provides the pose estimation at the current time (pose by events), while the mapping thread reconstructs a local point cloud map using the pose, event data, and time-surface image at that moment. This 3D point cloud is then used by the tracking thread for subsequent pose estimation.

#### 3.3.1. The 3D Point Cloud Reconstruction

The key to 3D point cloud reconstruction is estimating the depth of each event in the camera frame, and then combining the event’s coordinate with the depth to generate the corresponding point cloud. Assuming that we have time-surfaces ($\Gamma_{left}\left(\cdot ,t\right),\Gamma_{right}\left(\cdot ,t\right)$) at time *t* from left and right event cameras, respectively, the left event camera follows a camera trajectory $T_{t-\delta t:t}$. The inverse depth $\rho^{*}$ of an event $\left(e_{t-\epsilon }=\left(x,t-\epsilon \right),\epsilon \in \left[0,\delta t\right]\right)$ on the left event camera image plane can be estimated by the following function:

(2) $$\rho^{*}=\underset{\rho }{argmin}C\left(x,\rho ,\Gamma_{left}\left(\cdot ,t\right),\Gamma_{right}\left(\cdot ,t\right),T_{t-\delta t:t}\right)$$

(3) $$C≐\sum_{x_{1,i}\in W_{1},x_{2,i}\in W_{2}}r_{i}^{2}\left(\rho \right)$$

The residual

(4) $$r_{i}\left(\rho \right)≐\Gamma_{left}\left(x_{1,i},t\right)-\Gamma_{right}\left(x_{2,i},t\right)$$

denotes the difference of the temporal between two corresponding pixels $x_{1,i}$ and $x_{2,i}$ inside neighborhoods (i.e., patches) $W_{1}$ and $W_{2}$, and $x_{1}$ and $x_{2}$ are the centers of $W_{1}$ and $W_{2}$, respectively. The points $x_{1}$ and $x_{2}$ are given by

(5) $$\begin{array}{cc} x_{1} & =\pi {(}^{c_{t}}T_{c_{t-\epsilon }}\cdot \pi^{-1}\left(x,\rho_{k}\right))\\ x_{2} & =\pi {(}^{right}T_{left}\cdot^{c_{t}}T_{c_{t-\epsilon }}\cdot \pi^{-1}\left(x,\rho_{k}\right)) \end{array}$$

The function π projects a 3D point in the space onto the camera’s imaging plane, and $\pi^{-1}$ back-projects a pixel into 3D space with inverse depth ρ. Tleftright is the transformation matrix from the left event camera to the right event camera. Through the above explanation, we can estimate the depth of each event point. Suppose that the depth of each event point is known. Then, we fuse all inverse depth estimates to obtain a more accurate semi-dense 3D map of the current moment, which will be used for tracking later.

#### 3.3.2. Pose Estimation by Events

**Figure 4:**
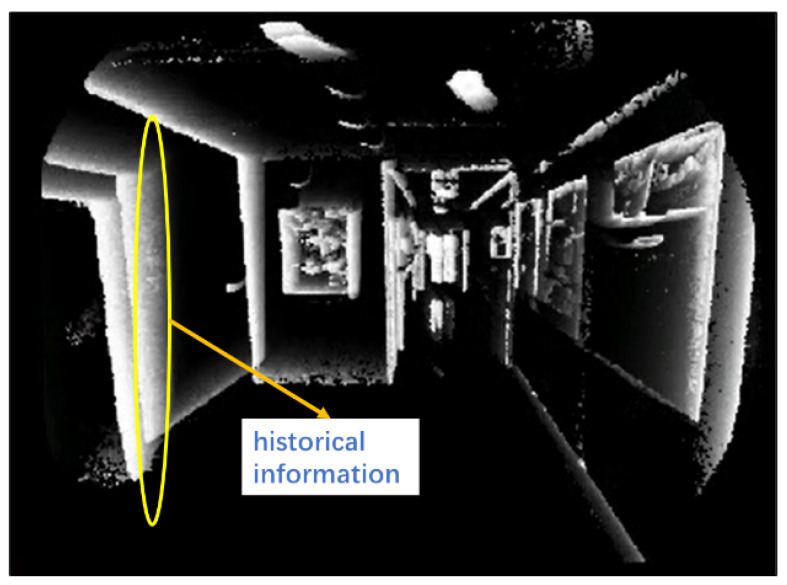
Time-surface and its included historical information.

In this section, we explain how to obtain the current pose using the visual method. Time-surfaces contain edge information in the scene (as shown in Figure 4). Historical information records the camera’s historical movements. Large values correspond to events that are close to the current time. Since in the image, a grayscale value of 255 represents white and 0 represents black. To construct a minimum optimization problem, we use the time-surface negative rather than the time-surface, defined by the following.

(6) $$\bar{\Gamma }\left(x,t\right)=1-\Gamma \left(x,t\right)$$

$\bar{\Gamma }\left(x,t\right)$ is also rescaled to the range [0,255]. The tracking problem can be formulated as follows. Let $S^{F_{ref}}=\left\{x_{i}\right\}$ be a set of pixel locations with valid inverse depth $\rho_{i}$ in the reference frame $F_{ref}$. Assuming the TS negative at time k is known, obtained by ${\bar{\Gamma }}_{left}\left(\cdot ,k\right)$, we need to find the pose *T*, which makes the warped semi-dense map $T(S^{F_{ref}})$ align well with ${\bar{\Gamma }}_{left}\left(\cdot ,k\right)$. In an ideal scenario, the 3D points in space should be projected onto pixels with a grayscale value of 0 in the TS negative image. This problem can be defined as

(7) $$\psi^{*}=\underset{\psi }{argmin}\sum_{x\in S^{F_{ref}}}{\left({\bar{\Gamma }}_{left}\left(W\left(x,\rho ;\psi \right),k\right)\right)}^{2}$$

where the warping function

(8) $$W\left(x,\rho ;\psi \right)≐\pi_{left}\left(T\left(\pi_{ref}^{-1}\left(x,\rho \right),G\left(\psi \right)\right)\right)$$

transfers points from $F_{ref}$ to the current frame. ψ is a 6×1 vector representing rotation and translation. The function G(ψ) provides the matrix form of ψ. The function $\pi_{ref}^{-1}\left(\cdot \right)$ back-projects a pixel x into space with the inverse depth ρ, while $\pi_{left}\left(\cdot \right)$ projects a space point onto the image plane of the left event camera. T(·) transforms a 3D point with motion G(ψ) from $F_{ref}$ to the left frame $F_{k}$. Based on the above explanation, we can obtain the pose ψ by the visual method.

### 3.4. ESKF Description

#### 3.4.1. Structure of the ESKF State Vector

The goal of the proposed filter is to track the pose of the IMU frame {I} (generally considered as body frame) with respect to a global frame of reference {G}. An overview of the algorithm is given in Algorithm 1. The IMU data are processed for propagating the ESKF state and covariance. Then, each time an observation is received, the state vector is updated. The IMU state is a 15×1 vector which is defined as

(9) $$x_{IMU}={\left(p^{T},v^{T},q^{T},b_{a}^{T},b_{\omega }^{T}\right)}^{T}$$

where the quaternion q represents the rotation from the frame {I} to the frame {G}. The vectors $v\in R^{3}$ and $p\in R^{3}$ represent the velocity and position of the body frame {I} in the global frame {G}. The vectors $b_{a}\in R^{3}$ and $b_{\omega }\in R^{3}$ are the biases of the linear acceleration and angular velocity from the IMU, which are modeled as random walk processes, driven by the white Gaussian noise vectors $n_{ba}$ and $n_{b\omega }$, respectively. Following (9), the IMU error-state is defined as

(10) $$\delta x_{IMU}={\left(\delta p^{T},\delta v^{T},\delta q^{T},\delta b_{a}^{T},\delta b_{\omega }^{T}\right)}^{T}$$

the standard error definition is used for the position, velocity, and biases (e.g., $\tilde{p}=p+\delta p$, $\tilde{p}$ is the real value of the position, and p is the ideal value). For the quaternion, a different error definition is employed, which is defined by the relation $\tilde{q}=q⨂\delta q$. The symbol ⨂ denotes quaternion multiplication. The error quaternion is

(11) $$\delta q=\left[\begin{array}{c} 1\\ \frac{\delta \theta }{2} \end{array}\right]$$

where δθ represents a small angle rotation.
**Algorithm 1** Framework of SEVIO**Input:**  The event data from two event-based cameras;  The acceleration and angular velocity from IMU. **Output:**  The pose of the body frame {I} with respect to the global frame {G}.
1:Initialize: A modified SGM method (vision); Estimation of IMU biases and the gravity direction (ESKF).2:IMU propagation;3:**if** no observation **then**4:    Posterior equals prior (Equation (20))5:**else**6:    Update posterior pose (Equation (21))7:    Clear the error-state (Equation (22))8:**end if**


#### 3.4.2. Process Model

In this section, we will explain how to update the error state variable δx by IMU data. The continuous dynamics of the IMU error-state is

(12) $$\begin{array}{cc} \delta \dot{p} & =\delta v\\ \delta \dot{v} & =-R_{t}{\left[a_{t}-b_{a_{t}}\right]}_{\times }\delta \theta +R_{t}\left(n_{a}-\delta b_{a}\right)\\ \delta \dot{\theta } & =-{\left[\omega_{t}-b_{\omega_{t}}\right]}_{\times }\delta \theta +n_{\omega }-\delta b_{\omega }\\ \delta \dot{b_{a}} & =n_{ba}\\ \delta \dot{b_{\omega }} & =n_{b\omega } \end{array}$$

In these expressions, $a_{t}$ and $\omega_{t}$ are the acceleration and angular velocity from IMU measurements. ${\left[\cdot \right]}_{\times }$ means the skew symmetric matrix. $R_{t}$ is the rotation matrix from frame {I} to frame {G}, $n_{a}$ and $n_{\omega }$ are the zero-mean, white Gaussian noise processes modeling the measurement noise. The linearized continuous-time model for the IMU error-state is

(13) $$\delta \dot{x}=F_{t}\delta x+B_{t}n$$

where $n={\left(n_{a}^{T},n_{\omega }^{T},n_{b_{a}}^{T},n_{b_{\omega }}^{T}\right)}^{T}.$ The vectors $n_{ba}$ and $n_{b\omega }$ are the random walk rate of the accelerometer and gyroscope measurement biases. $F_{t}$ and $B_{t}$ are shown in Appendix A.1.

To deal with the discrete time measurement from the IMU, we apply the median integral to propagate the estimated IMU state:

(14) $$\begin{array}{cc} q_{\omega b_{k}} & =q_{\omega b_{k-1}}⊗\left[\begin{array}{c} \cos\frac{\phi }{2}\\ \frac{\phi }{\phi }\sin\frac{\phi }{2} \end{array}\right]\\ v_{k} & =v_{k-1}+\left(\frac{R_{\omega b_{k}}a_{k}+R_{\omega b_{k-1}}a_{k-1}}{2}-g\right)\left(t_{k}-t_{k-1}\right)\\ p_{k} & =p_{k-1}+\frac{v_{k}+v_{k-1}}{2}\left(t_{k}-t_{k-1}\right) \end{array}$$

where $\phi =\frac{\omega_{k-1}+\omega_{k}}{2}\left(t_{k}-t_{k-1}\right)$, ϕ=∥ϕ∥.

To discretize (13),

(15) $$\delta x_{k}=F_{k-1}\delta x_{k-1}+B_{k-1}n_{k}$$

where $F_{k-1}$ and $B_{k-1}$ are shown in Appendix A.2.

#### 3.4.3. Measurement Model

We use the pose obtained through the visual method as the observation. Now we introduce the measurement model employed for updating the state estimates. Generally, the observation equation is written as

(16) $$y=G_{t}\delta x+C_{t}n_{R}$$

In this expression, $G_{t}$ is the measurement Jacobian matrix, and $n_{R}$ is the observation noise:

(17) $$n_{R}={\left(n_{\delta {\bar{p}}_{x}},n_{\delta {\bar{p}}_{y}},n_{\delta {\bar{p}}_{z}},n_{\delta {\bar{\theta }}_{x}},n_{\delta {\bar{\theta }}_{y}},n_{\delta {\bar{\theta }}_{z}}\right)}^{T}$$

The noise term $C_{t}n_{R}$ is zero-mean, white, and uncorrelated to the state for the ESKF framework to be applied. In this application condition, the observation equation is

(18) $$y={\left[\delta {\bar{p}}^{T},\delta {\bar{\theta }}^{T}\right]}^{T}$$

According to the previous formulas, we obtain the equations of discrete ESKF, which are shown in Appendix A.3.

#### 3.4.4. Fuse Poses from IMU and Vision

In the preceding sections, we present the process model and measurement model. Now we introduce in detail the updating phase of the ESKF. We will fuse the pose obtained from the IMU with the pose obtained from vision to generate the fused pose, which serves as the output pose of our system. The whole process is summarized in Algorithm 1.

Before we start the ESKF process, we need to know the initial state of the system. In our implementation, we assume that the initial state of the system is static and take the average value of IMU output for a period of time to estimate the gravity direction and the biases of the gyroscope and accelerometer. Then, we obtain the rotation from the IMU frame to the world. Next is initializing the filter parameters, including state vector $\delta {\hat{x}}_{0}$, variance ${\hat{P}}_{0}$, process noise Q, and observation noise R. The specific form is shown in Appendix A.4.

Then, we integrate the IMU output according to (14) for performing filter prior estimate and executing the first two steps of the five steps of the ESKF:

(19) $$\begin{array}{cc} \delta {\overset{ˇ}{x}}_{k} & =F_{k-1}\delta {\hat{x}}_{k-1}+B_{k-1}n_{k}\\ {\overset{ˇ}{P}}_{k} & =F_{k-1}{\hat{P}}_{k-1}F_{k-1}^{T}+B_{k-1}Q_{k}B_{k-1}^{T} \end{array}$$

When there is no observation, the posterior equals the prior:

(20) $$\begin{array}{cc} \delta {\hat{x}}_{k} & =\delta {\overset{ˇ}{x}}_{k}\\ {\hat{P}}_{k} & ={\overset{ˇ}{P}}_{k}\\ {\hat{x}}_{k} & ={\overset{ˇ}{x}}_{k} \end{array}$$

When the mapping thread of vision module works, we consider that an observation is received and execute the last three steps of the five steps of the ESKF:

(21) $$\begin{array}{cc} K_{k} & ={\overset{ˇ}{P}}_{k}G_{k}^{T}{\left(G_{k}{\overset{ˇ}{P}}_{k}G_{k}^{T}+C_{k}R_{k}C_{k}^{T}\right)}^{-1}\\ {\hat{P}}_{k} & =\left(I-K_{k}G_{k}\right){\overset{ˇ}{P}}_{k}\\ \delta {\hat{x}}_{k} & =\delta {\overset{ˇ}{x}}_{k}+K_{k}\left(y_{k}-G_{k}\delta {\overset{ˇ}{x}}_{k}\right) \end{array}$$

After that, we update the posterior pose and clear the state vector $\delta {\hat{x}}_{k}$:

(22) $$\begin{array}{cc} {\hat{p}}_{k} & ={\overset{ˇ}{p}}_{k}-\delta {\hat{p}}_{k}\\ {\hat{v}}_{k} & ={\overset{ˇ}{v}}_{k}-\delta {\hat{v}}_{k}\\ {\hat{R}}_{k} & ={\overset{ˇ}{R}}_{k}\left(I-{\left[\delta {\hat{\theta }}_{k}\right]}_{\times }\right)\\ {\hat{b}}_{ak} & ={\overset{ˇ}{b}}_{ak}-\delta {\hat{b}}_{ak}\\ {\hat{b}}_{\omega k} & ={\overset{ˇ}{b}}_{\omega k}-\delta {\hat{b}}_{\omega k} \end{array}$$

## 4. Experiments

In this section, we present the datasets that we used and evaluate the proposed stereo event-based VIO system. The results show that SEVIO produces more accurate trajectories than ESVO and U-SLAM. Then, we illustrate the real-time performance of our pipeline on different resolution event data.

### 4.1. Datasets Used

We use public datasets to evaluate our stereo VIO system [39,40]. The datasets from [39] were collected using a UAV, while the datasets from [40] were gathered using a mobile vehicle. Our method assumes that the undistorted coordinates have been obtained. The parameters of the camera and IMU, along with their extrinsic parameters, are provided in public datasets. Different datasets use different event cameras, and their parameters are listed in Table 1.

### 4.2. Accuracy Evaluation

**Figure 5:**
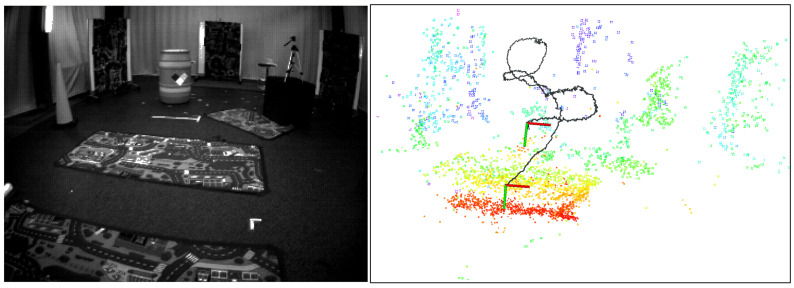
Algorithm performance. The left image shows the experimental scene, while the right image displays the local point clouds and trajectories.

We provide an example to present the performance of our algorithm in Figure 5. On the left is the scene of the dataset, while on the right is the partial output of the algorithm, including the local point cloud and trajectory. To demonstrate the performance of the stereo VIO system, we save the trajectories output by our algorithm and compare them with the ground truth provided by the dataset. We use a general approach to evaluate our pose estimates: relative pose error (RPE) and absolute pose error (APE). We compare our method with two approaches: one is ESVO [16], which demonstrates the improvement of our approach over pure vision, and the other is U-SLAM (event camera+IMU) [28,29], which highlights the advantages of our approach compared to other event camera-based VIO systems.

**Figure 6:**
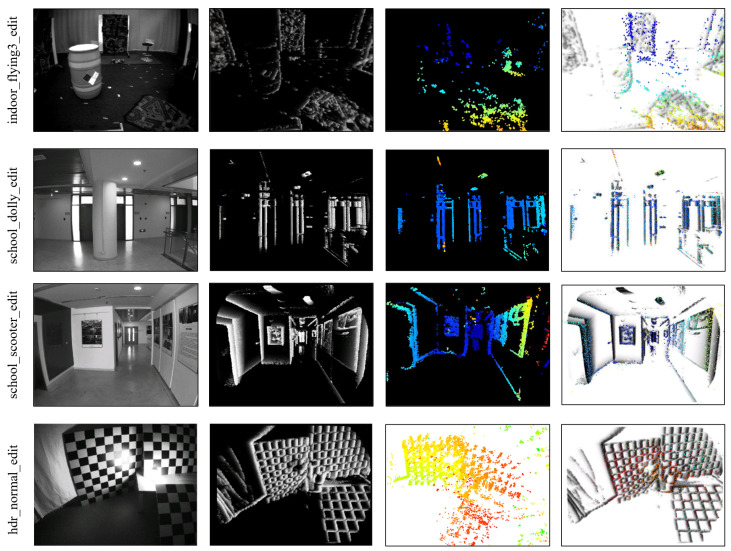
The first column shows images from a traditional camera. The second column is the time-surface. The third column is the inverse depth map. The last column is the warping depth map overlaid on the time-surface negative.

The evaluation results for each sequence are listed in Table 2, and the best results in each sequence are bolded. It shows that most of the trajectory accuracy is improved. Some algorithm results are shown in Figure 6. Our method is less accurate than ESVO on *indoor_flying1_edit*. In an ideal situation, visual initialization should occur immediately after IMU initialization. However, due to the lack of texture in the scene, the conditions for visual initialization may not be met. In our example, once IMU initialization is completed, the drone begins to move, but the lack of scene information prevents visual initialization. After a delay, the visual initialization is eventually completed, but by that time, the bias of IMU is different from that during the IMU initialization. In addition, better vision and filter parameters may further improve the accuracy of the results. Furthermore, it is evident that our method outperforms U-SLAM in both accuracy and robustness.

One of the advantages of event cameras is that they can work in HDR scenes. Event cameras have higher dynamic range (with about 140 dB) than tradition cameras (with about 60 dB). We test this feature of the event camera using a dataset *hdr_normal_edit*. It is collected in a dark room with a light source to increase the brightness range in the scene. This can result in overexposure in some areas of traditional camera images, while other areas may be too dark to capture scene details. In this challenging scene, the performance of the event camera is not affected.

### 4.3. Real-Time Performance

We test the real-time performance of the algorithm on a standard CPU (Intel Xeon Platinum 8375C CPU @ 2.90 GHz). Our system can work in real-time with event data from low-resolution cameras (e.g., 346×260). For higher-resolution cameras (e.g., 640×480), we need to reduce the play speed of the data for better performance (e.g., 0.2). This is because compared to traditional cameras, within the same resolution and time period, event cameras capture data with microsecond-level delays. Although they only record edge information of the scene, they still generate a large volume of data, leading to significant computational power consumption. Choosing partly appropriate event data to participate in calculations may be a solution.

## 5. Conclusions

In this paper, we presented a stereo event-based visual–inertial pipeline using a ESKF framework. Pose estimation is essential for robot control. Due to the hardware advantages of event cameras, they have the potential to handle challenging scenes more effectively than traditional cameras. Compared with the visual odometry for stereo event cameras (ESVO), our method improves accuracy by approximately 30%. Compared to the event camera-based visual–inertial fusion method U-SLAM, our approach also demonstrates superior accuracy and robustness. We also demonstrate the performance of event cameras in high dynamic environments. Multi-sensor fusion is the future direction for pose estimation, and event cameras can serve as a valuable supplement to traditional cameras in the field of robotics. In the future work, since event cameras generate a larger volume of data compared to traditional cameras, further research is needed on how to filter event camera data to reduce computational complexity.

## Figures and Tables

**Table 1:** Parameters of event camera used in the datasets.

Dataset	Camera	IMU	Resolution (pixel)	Baseline (cm)
MVSEC	DAVIS 346	MPU6150	346×260	10.0
VECtor	Prophesee Gen3	MTi-30	640×480	17.0

**Table 2:** Absolute pose error and relative pose error (RMSE).

Sequences	ESVO		U-SLAM		SEVIO (Ours)	
	APE (m)	RPE (m)	APE (m)	RPE (m)	APE (m)	RPE (m)
indoor_flying1_edit	**0.190**	0.014	-	-	0.299	**0.011**
indoor_flying3_edit	0.342	0.027	0.473	0.024	**0.266**	**0.010**
school_dolly_edit	0.990	0.077	1.260	0.082	**0.703**	**0.075**
school_scooter_edit	2.666	0.233	-	-	**1.291**	**0.195**
units_dolly_edit	0.714	0.096	0.628	0.089	**0.514**	**0.084**
units_scooter_edit	0.652	0.083	-	-	**0.461**	**0.072**
sofa_normal_edit	0.368	0.031	-	-	**0.286**	**0.024**
desk_normal_edit	0.416	0.034	0.752	0.048	**0.325**	**0.027**
hdr_normal_edit	0.157	0.012	-	-	**0.126**	**0.009**

## Data Availability

The original contributions presented in the study are included in the article.

## Appendix A

### Appendix A.1

The $F_{t}$ and $B_{t}$ in (13)

$$F_{t}=\left[\begin{array}{ccccc} 0_{3\times 3} & I_{3} & 0_{3\times 3} & 0_{3\times 3} & 0_{3\times 3}\\ 0_{3\times 3} & 0_{3\times 3} & -R_{t}{\left[a_{t}-b_{a_{t}}\right]}_{\times } & -R_{t} & 0_{3\times 3}\\ 0_{3\times 3} & 0_{3\times 3} & -{\left[\omega_{t}-b_{\omega_{t}}\right]}_{\times } & 0_{3\times 3} & -I_{3}\\ 0_{3\times 3} & 0_{3\times 3} & 0_{3\times 3} & 0_{3\times 3} & 0_{3\times 3}\\ 0_{3\times 3} & 0_{3\times 3} & 0_{3\times 3} & 0_{3\times 3} & 0_{3\times 3} \end{array}\right]$$

$$B_{t}=\left[\begin{array}{cccc} 0_{3\times 3} & 0_{3\times 3} & 0_{3\times 3} & 0_{3\times 3}\\ R_{t} & 0_{3\times 3} & 0_{3\times 3} & 0_{3\times 3}\\ 0_{3\times 3} & I_{3} & 0_{3\times 3} & 0_{3\times 3}\\ 0_{3\times 3} & 0_{3\times 3} & I_{3} & 0_{3\times 3}\\ 0_{3\times 3} & 0_{3\times 3} & 0_{3\times 3} & I_{3} \end{array}\right]$$

### Appendix A.2

The $F_{k-1}$ and $B_{k-1}$ in (15)

$$F_{k-1}=I_{15}+F_{t}T$$

$$B_{k-1}=\left[\begin{array}{cccc} 0_{3\times 3} & 0_{3\times 3} & 0_{3\times 3} & 0_{3\times 3}\\ R_{k-1}T & 0_{3\times 3} & 0_{3\times 3} & 0_{3\times 3}\\ 0_{3\times 3} & I_{3}T & 0_{3\times 3} & 0_{3\times 3}\\ 0_{3\times 3} & 0_{3\times 3} & I_{3}\sqrt{T} & 0_{3\times 3}\\ 0_{3\times 3} & 0_{3\times 3} & 0_{3\times 3} & I_{3}\sqrt{T} \end{array}\right]$$

T is the period of the ESKF.

### Appendix A.3

Discrete ESKF equations

$$\begin{array}{cc} \delta {\overset{ˇ}{x}}_{k} & =F_{k-1}\delta {\hat{x}}_{k-1}+B_{k-1}n_{k}\\ {\overset{ˇ}{P}}_{k} & =F_{k-1}{\hat{P}}_{k-1}F_{k-1}^{T}+B_{k-1}Q_{k}B_{k-1}^{T}\\ K_{k} & ={\overset{ˇ}{P}}_{k}G_{k}^{T}{\left(G_{k}{\overset{ˇ}{P}}_{k}G_{k}^{T}+C_{k}R_{k}C_{k}^{T}\right)}^{-1}\\ {\hat{P}}_{k} & =\left(I-K_{k}G_{k}\right){\overset{ˇ}{P}}_{k}\\ \delta {\hat{x}}_{k} & =\delta {\overset{ˇ}{x}}_{k}+K_{k}\left(y_{k}-G_{k}\delta {\overset{ˇ}{x}}_{k}\right) \end{array}$$

### Appendix A.4

Variance, process noise, and observation noise

Variance:

$${\hat{P}}_{0}=\left[\begin{array}{ccccc} P_{\delta p} & 0_{3\times 3} & 0_{3\times 3} & 0_{3\times 3} & 0_{3\times 3}\\ 0_{3\times 3} & P_{\delta v} & 0_{3\times 3} & 0_{3\times 3} & 0_{3\times 3}\\ 0_{3\times 3} & 0_{3\times 3} & P_{\delta \theta } & 0_{3\times 3} & 0_{3\times 3}\\ 0_{3\times 3} & 0_{3\times 3} & 0_{3\times 3} & P_{\delta b_{a}} & 0_{3\times 3}\\ 0_{3\times 3} & 0_{3\times 3} & 0_{3\times 3} & 0_{3\times 3} & P_{\delta b_{\omega }} \end{array}\right]$$

Process noise and observation noise:

$$Q=\left[\begin{array}{cccc} Q_{a} & 0_{3\times 3} & 0_{3\times 3} & 0_{3\times 3}\\ 0_{3\times 3} & Q_{\omega } & 0_{3\times 3} & 0_{3\times 3}\\ 0_{3\times 3} & 0_{3\times 3} & Q_{b_{a}} & 0_{3\times 3}\\ 0_{3\times 3} & 0_{3\times 3} & 0_{3\times 3} & Q_{b_{\omega }} \end{array}\right]$$

$$R=\left[\begin{array}{cc} R_{\delta p} & 0_{3\times 3}\\ 0_{3\times 3} & R_{\delta \theta } \end{array}\right]$$
